# Immunological Biomarkers to Assess Activity and Treatment Response in IgG4-Related Disease

**DOI:** 10.3390/medicina62020323

**Published:** 2026-02-04

**Authors:** Patricia Moya-Alvarado, Marta Lopez-Gomez, Laura Martínez-Martinez, Hye Sang Park, Teresa Franco Leyva, Mar Concepción Martín, Helena Codes-Mendez, Anna Calvet Lacruz, Sara Calleja, Berta Magallares, Iván Castellví, Antonio J. Barros-Membrilla, Julia Bernárdez, Hèctor Corominas

**Affiliations:** 1 Rheumatology Department, Hospital de la Santa Creu i Sant Pau, 08025 Barcelona, Spain; pmoyaa@santpau.cat (P.M.-A.); hsang@santpau.cat (H.S.P.); hcodes@santpau.cat (H.C.-M.); bmagallares@santpau.cat (B.M.); icastellvi@santpau.cat (I.C.); juliabernardez8@gmail.com (J.B.); hcorominas@santpau.cat (H.C.); 2 Department of Medicine, Universitat Autònoma de Barcelona (UAB), 08193 Barcelona, Spain; mlopezgom@csi.cat; 3 Multiorgan Damage and Rheumatology Research Group, Institut de Recerca Sant Pau (IR SANT PAU), 08041 Barcelona, Spain; 4 Rheumatology Department, Complex Hospitalari Universitari Moisès Broggi, 08970 Sant Joan Despí, Spain; 5 Immunology Department, Hospital de la Santa Creu i Sant Pau, IIB Sant Pau, Universitat Autònoma de Barcelona, 08025 Barcelona, Spain; tfrancol@santpau.cat (T.F.L.); acalvetl@santpau.cat (A.C.L.); scalleva7@alumnes.ub.edu (S.C.); 6 Gastroenterology Department, Hospital de la Santa Creu i Sant Pau, 08025 Barcelona, Spain; mconcepcion@santpau.cat; 7 Cardiology Department, Hospital de la Santa Creu i Sant Pau, 08025 Barcelona, Spain; abarros@santpau.cat

**Keywords:** IgG4-related disease, disease activity, Responder Index, IgG4, serology, biomarkers, cytokines, immune profiling, PET/CT, treatment

## Abstract

*Background and Objectives*: IgG4-related disease is a chronic fibro-inflammatory condition. Despite the development of classification and responder indexes, reliable biomarkers for disease activity and therapeutic monitoring remain limited. We evaluate the performance of a panel of biomarkers, including cytokine profiles, plasmablasts and conventional markers. *Materials and Methods*: We conducted a cross-sectional, single-center study, involving 35 patients diagnosed with IgG4-RD. Disease activity was evaluated using the IgG4-RD Responder Index (RI), Damage Index (DI) and clinical assessment. Laboratory evaluation included serum IgG4, total IgG, CRP, ESR, eosinophils, IgE, complement levels, and cytokine profiling via multiplex immunoassay. B cell subpopulations were analyzed by flow cytometry. Statistical analyses were performed using STATA/BE 17.0. *Results*: Patients with active disease (RI > 4 or clinical judgment) exhibited significantly higher levels of total IgG (*p* = 0.02), IgG4 (*p* = 0.01), and IL-5 (*p* = 0.03). PET-positive patients showed a Th1-skewed immune profile, with elevated IFN-γ/IL-4 (*p* < 0.001), reduced IL-21/IFN-γ (*p* = 0.03), and increased eosinophils (*p* = 0.03). Clinician-assessed active disease was associated with higher total IgG levels (*p* = 0.01). Treatment-specific effects were observed: prednisone was associated with lower IgG4 and C3 levels. Notably, plasmablasts did not consistently correlate with clinical or imaging activity scores, possibly reflecting treatment status or B cell dynamics. *Conclusions*: This study demonstrates that cytokine ratios, particularly those involving IL-5, IL-13, IL-21, and IFN-γ, offer complementary information to traditional serological markers for IgG4-RD activity. While PET/CT-defined activity was best reflected by biomarkers of an IFN-γ-mediated pathway, the IgG4-RD RI demonstrated a stronger association with conventional humoral markers like serum IgG4 and total IgG. None of these biomarkers correlated with organ damage.

## 1. Introduction

IgG4-related disease (IgG4-RD) is an idiopathic, chronic, immune-mediated fibro-inflammatory condition characterized by tissue and organ infiltration by T and B lymphocytes and IgG4-secreting plasma cells, often with mass-forming lesions [1]. It can affect virtually any organ system and, if left untreated, may lead to progressive fibrosis, irreversible organ dysfunction, and death [1]. Recognized as a distinct clinical entity only within the past decade, IgG4-RD predominantly affects middle-aged to elderly men and is more frequently reported in Asian and Caucasian populations [2,3]. However, its true prevalence is likely underestimated because its subtle, heterogeneous presentation often mimics a wide spectrum of immune, autoimmune, and inflammatory disorders [3].

Although nearly any organ can be affected, four predominant clinical phenotypes have been described based on the distribution of organ involvement [2,4,5,6]: pancreatic–hepatobiliary disease (31% of cases), retroperitoneal fibrosis with or without aortitis (24%), head and neck-limited disease (24%), and Mikulicz-like syndrome with systemic involvement (22%).

The pathogenesis of organ damage and progressive fibrosis in IgG4-RD is complex and not fully understood, but current evidence indicates a dysregulated immune response involving both innate and adaptive immunity, driven by antigen-specific activation of B and T lymphocytes [7,8,9]. IgG4-RD was initially thought to result from a Th2-skewed CD4+ response promoting B cell class-switching to IgG4, a subclass unable to cross-link antigens, form immune complexes, or activate the classical complement cascade [7]. Newer evidence has demonstrated a more complex immunopathology involving the presence of cytotoxic CD4+ T lymphocytes (CD4+ CTLs), regulatory T cells (Tregs), follicular helper T (Tfh) cells, and memory B cells within affected tissues [7,8,9,10]. Interactions with follicular dendritic cells and cytokines such as interleukin (IL)-4, IL-10, and IL-21 facilitate somatic hypermutation, class-switching to IgG4, and affinity maturation [11].

Traditional biomarkers include a range of serological and cellular markers that reflect the underlying immune dysregulation and inflammation [2,10,11,12]. The most widely used is serum IgG4, which is elevated in approximately 60–70% of patients; however, it lacks specificity, and normal levels do not exclude the diagnosis [2,10,12]. Circulating plasmablasts (activated B cells) are often markedly elevated in active disease and decline with effective B cell depleting therapies such as rituximab, making them a dynamic and more specific biomarker [2,10,12,13]. Other findings include peripheral eosinophilia and increased IgE, reflecting the Th2-skewed immune response [10,11,12,13,14] reduced complement levels (C3 and C4) in systemic or renal involvement; and mildly elevated acute-phase reactants such as erythrocyte sedimentation rate (ESR) and C-reactive protein (CRP) [12]. Further, cytokine networks, particularly ILs and interferon-gamma (IFN-γ), play a key role in shaping the immune response and fibrosis in IgG4-RD [10,11,12].

Diagnosing IgG4-RD remains challenging due to the lack of a definitive biomarker. In 2012, Umehara and Okazaki proposed diagnostic criteria combining typical clinical features, elevated serum IgG4 and characteristic histopathology, applicable only after excluding malignancy and mimics and potentially less sensitive when biopsy is not feasible [15]. In 2019, ACR/EULAR introduced consensus classification criteria using a three-step approach: entry, exclusion criteria, and a weighted scoring system with a threshold ≥ 20 based on clinical findings, serum IgG4, imaging, and histopathology [16].

Given its potential for irreversible organ damage if untreated, timely diagnosis and treatment of IgG4-RD is essential. International consensus guidelines recommend histologic confirmation to rule out mimicking conditions and immediate therapy in active disease [17]. Glucocorticoids remain for first-line induction therapy, typically tapered after 2–4 weeks [17]. In cases of relapse or intolerance, immunosuppressive agents such as methotrexate or rituximab may be used, with the latter showing efficacy in refractory cases by depleting B cells and reducing IgG4-secreting plasmablasts, in some cases inducing complete remission [17,18,19]. Given the high relapse rate and the frequent need for long-term maintenance therapy, reliable biomarkers are essential to refine treatment algorithms, monitor therapeutic response, and improve long-term outcomes.

To address the lack of standardized disease activity measures, Carruthers et al. developed the IgG4-RD Responder Index (RI) in 2012 [20] which assigns a 0–4 score to each affected organ and serum IgG4 level to produce a composite activity score, with higher scores indicating greater activity. This tool correlates strongly with the physician’s global assessment [21] but it does not capture irreversible damage. To complement the RI, the Chinese IgG4-RD Consortium proposed the Damage Control Index (DI), assessing cumulative, long-term organ injury across 14 domains over more than 6 months [22]. Unlike RI, DI is unidirectional and reflects progressive disease rather than fluctuating inflammatory activity.

Fluorine-18 fluorodeoxyglucose positron emission tomography/computed tomography ([18F]-FDG PET/CT) has emerged as a particularly valuable tool in the diagnostic and therapeutic management of IgG4-RD [23]. Notably, [18F]-FDG PET/CT-derived volumetric parameters correlate with serum IgG4 levels and inflammatory biomarkers, offering an objective measure of disease activity, response to therapy, and distinction between active inflammation and residual fibrosis, often outperforming serological markers [23].

Despite available tools, traditional biomarkers have limited specificity and sensitivity in capturing the heterogeneity of IgG4-RD. In this context, the identification of novel biomarkers has become increasingly important to enhance diagnostic accuracy, monitor disease activity and therapeutic response, predict relapse, and monitor fibrosis progression [12].

Given these limitations, the objective of this study was to evaluate the clinical utility of novel immunological biomarkers in comparison with conventional measures. By analyzing these biomarkers across different disease phenotypes and stages of disease, we aim to determine their value to improve diagnostic accuracy, refine disease activity stratification, and support more personalized treatment monitoring. Ultimately, this study seeks to help refine the development of biomarker-guided strategies for the management of IgG4-RD.

## 2. Materials and Methods

### 2.1. Study Design and Population

A retrospective cross-sectional, single-center study was conducted at the Hospital de la Santa Creu i Sant Pau in Barcelona (HSCSP; Spain) involving 35 patients diagnosed with IgG4-RD between October 2022 and December 2024. All patients were diagnosed according to the comprehensive diagnostic criteria proposed by Umehara and Okazaki or the 2019 ACR/EULAR consensus classification criteria [1,16].

All cases were anonymized. The study was conducted following the guidelines of the Declaration of Helsinki and approved by the Ethics Committee of HSCSP (IIBSP-EIG-2022-92).

### 2.2. Clinical Data

Demographic and clinical data, including age at diagnosis, sex, number and type of organs involved, histopathological confirmation, and treatment received (prednisone and/or methotrexate and/or rituximab) were retrospectively collected from medical records. Patients were stratified into clinical phenotypes according to the Wallace classification based on organ involvement: pancreato-biliary, retroperitoneum and aorta, systemic Mikulicz, and head and neck-limited disease [4]. Detailed organ-specific findings were recorded, including imaging features such as pancreatic enlargement, renal cortical lesions, retroperitoneal and vascular involvement, and thoracic abnormalities. Histological findings were recorded when available, including the presence of IgG4-positive plasma cells, lymphocytic infiltration, storiform fibrosis, and obliterative phlebitis.

Disease activity was assessed using the IgG4-RD RI [20], with a score ≥ 4 considered indicative of active disease, and cumulative organ damage was evaluated using the mean Damage Index (DI) [22]. The presence of active disease was also determined based on clinician assessment at the time of data collection. Finally, imaging data included [18F]-FDG PET and contrast-enhanced CT findings. [18F]-FDG PET/CT scans were reviewed and classified as positive or negative based on visual assessment by nuclear medicine specialists. A scan was considered positive if abnormal [18F] FDG uptake.

### 2.3. Laboratory Variables

Routine laboratory assessments included peripheral blood eosinophil count, ESR, and the following parameters: serum total IgG and CRP, both measured using turbidimetry (Alinity, Abbott, Chicago, IL, USA); serum IgG4 and complement C3 and C4, measured by turbidimetry (Optilite, The Binding Site, Birmingham, UK); and total IgE quantified using fluoroimmunoassay (ImmunoCAP, Thermo Fisher Scientific, Uppsala, Sweeden).

### 2.4. Cytokine Profiling

Cytokine expression profiles were assessed in peripheral blood samples using a multiplex bead-based assay (HSTCMAG-18SK, Merck Millipore, Darmstadt, Germany), enabling the simultaneous quantification of multiple cytokines. Seven cytokines were selected based on their relevance to IgG4-RD immunopathogenesis: IL-1β, IL-10, IL-13, IFN-γ, IL-5, IL-4, and IL-21 [7,8,9,10,11]. In addition to analyzing absolute cytokine concentrations, we also considered the following specific cytokine ratios to capture the balance of immune responses: INF-γ/IL-10, IL-13/INF-γ, INF-γ/IL-1β, IL-21/INF-γ, INF-γ/IL-4, INF-γ/IL-5, IL-13/IL-10, IL-10/IL-1β, IL-21/IL-10, IL-10/IL-4, IL-10/IL-5, IL-13/IL-1β, IL-13/IL-21, IL-13/IL-4, IL-13/IL-5, IL-21/IL-1β, IL-1β/IL-4, IL-1β/IL-5, IL-21/IL-4, IL-21/IL-5, and IL-4/IL-5.

### 2.5. B Cell Subpopulations

B cell subsets were analyzed by flow cytometry using a two-step staining protocol. Total blood B lymphocytes were first quantified with a commercial antibody panel (BD Biosciences, San Jose, CA, USA), and samples with more than 30 × 10^6^ B lymphocytes per liter were selected for further analysis. A custom-made antibody panel was used to identify major B cell subsets, including naïve (CD19^+^ IgD^+^ CD27^−^), pre-switch memory B cells (CD19^+^ IgD^+^ CD27^+^), switch memory B cells (CD19^+^ IgD^−^ CD27^+^), exhausted memory B cells (CD19^+^ IgD^−^ CD27^−^), CD19^low^ B cells (CD19^low^ CD27^+^), CD21^low^ B cells (CD19^+^ CD38^−^), non-switch B cells (CD19^+^ IgD^+^ IgM^+^ CD27^+^ CD38^−^), transitional B cells (CD19^+^ IgD^+^ IgM^+^ CD27^−^ CD38^+^ CD24^+^), and plasmablasts (CD19^+^ IgD^−^ IgM^−^ CD27^+^ CD38^+^).

### 2.6. Outcomes

The primary outcome was diagnostic utility, defined as the ability of routine laboratory parameters, cytokine profiles, and B cell subpopulations—plasmablasts—to discriminate between active and inactive disease, assessed by [18F]-FDG PET/CT findings (positive vs. negative), clinical judgment (active vs. inactive), the IgG4-RD RI (≤4 vs. >4 score), and to correlate with cumulative damage as measured by the mean IgG4-RD DI score. Secondary outcomes included the association of biomarker levels with the type of treatment received, specifically glucocorticoids.

### 2.7. Statistical Analysis

Descriptive statistics were used to summarize demographic, clinical, laboratory, histological, and imaging characteristics. Normality of data distribution was assessed using the Shapiro–Wilk test. Categorical variables were summarized as frequencies and percentages, and quantitative variables reported as mean (standard deviation, SD) or median with interquartile range, as appropriate. Comparisons of continuous variables between active and inactive disease groups were performed using an independent samples Student’s *t*-test or a Mann–Whitney U test, depending on the normality of the data distribution. For transparency, the results tables include a legend indicating the statistical test applied for each *p* value. For comparing a continuous variable across more than two groups, the Kruskal–Wallis test was employed. The correlation between biomarker levels and the IgG4-DI was analyzed using Spearman’s rank correlation coefficient (ρ). A *p*-value < 0.05 was considered statistically significant. Findings with *p*-values between 0.05 and 0.10 were interpreted as trends toward significance and were reported descriptively to highlight potentially relevant associations. All analyses were performed using STATA/BE 17.0, [24] and data visualizations were generated with R statistical software version 4.4.1 [25].

## 3. Results

### 3.1. Patient Characteristics and Phenotypic Distribution

A total of 35 patients with IgG4-RD were included in the study cohort, and the detailed characteristics are summarized in Table 1. The median age was 63 years (P25–P75, 52–72), and most patients were male (77.1%). All patients fulfilled at least one diagnostic criterion: 23 (65.7%) met the Okazaki criteria, 23 (65.7%) satisfied the ACR/EULAR classification system, and 32 (91.4%) were diagnosed based on expert clinical judgment.

An elevated serum IgG4 concentration was reported in 17 cases (48,57%). Organ enlargement was observed in almost all patients (94.3%), but only three (8.8%) exhibited glandular involvement affecting two or more glands. The most commonly involved organs were the retroperitoneum (34.3%), followed by the kidney (28.6%), the pancreato-biliary system (28.6%), and the thorax (20.0%). Based on organ involvement patterns (Wallace phenotypic classification), 21 patients (60.0%) had the retroperitoneum/aortic subtype, 8 patients (22.9%) the pancreato-biliary subtype, 5 patients (14.3%) the systemic Mikulicz subtype, and 1 (2.9%) head and neck-limited phenotype. The mean IgG4-RD DI was 3.09 (SD = 1.5), consistent with a modest to moderate degree of accumulated organ damage.

Systemic symptoms were relatively infrequent, with toxic syndrome in seven patients (20.0%) and fever in five (14.3%). Low back pain was reported in eight patients (22.9%).

### 3.2. Baseline Disease Activity and Imaging Findings

A detailed summary of baseline disease activity and imaging findings is provided in Table 1. At inclusion, 12 patients (34.3%) were classified as having clinically active disease based on physician judgment. Among the 28 patients who underwent [18F]-FDG-PET/CT scans, active metabolic lesions were identified in 8 cases (28.6%). The mean IgG4-RD RI was 4.83 (SD = 4.0), with 19 patients (54.3%) scoring ≥4, indicating active disease.

Abdominal CT showed aortic involvement in 14 patients (40.0%) and retroperitoneal soft tissue thickening in 6 (21.4%). Histological samples were available in 11 patients; of these, 10 (90.9%) showed dense plasmacytic infiltrates and 4 (36.4%) storiform fibrosis.

### 3.3. Biomarker Profiles According to [18F]-FDG-PET/CT Activity

**Figure 1:**
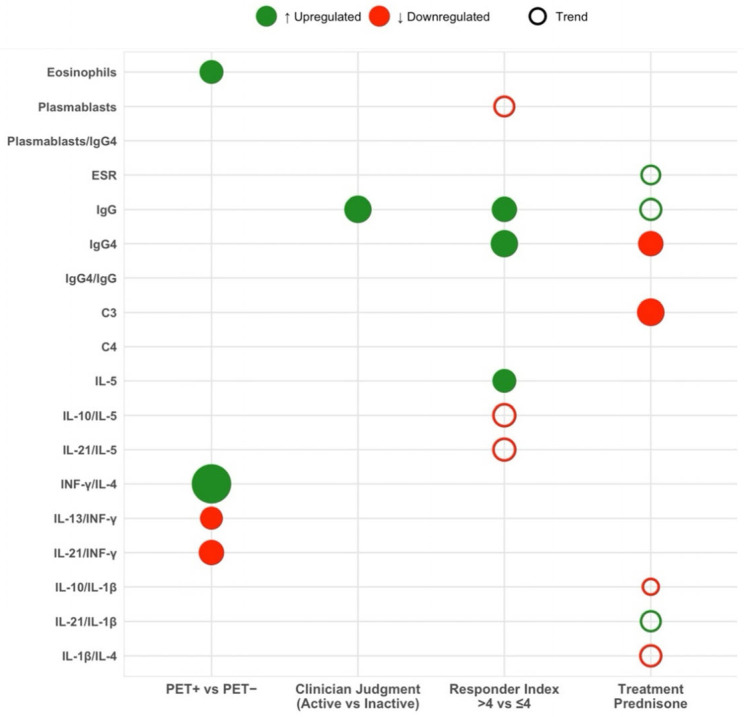
Bubble plot showing significant and near-significant biomarker differences across clinical contexts. Each circle represents a biomarker. Filled circles indicate statistically significant differences (*p* < 0.05) and non-filled circles denote trends toward significance (0.05 ≤ *p* < 0.10). Color denotes direction of change—green for upregulation and red for downregulation—and symbol size reflects the strength of the association (−log_10_ *p*-value).

Although most biomarkers did not differ between groups, eosinophil counts were significantly higher in PET-positive individuals compared to PET-negative counterparts (0.24 × 10^9^/L vs. 0.15 × 10^9^/L; *p* = 0.03) (Table 2; Figure 1). Furthermore, PET-positive patients had significantly higher IFN-γ/IL-4 ratios (3.46 vs. 0.98; *p* < 0.001) and lower IL-21/IFN-γ (0.03 vs. 0.07; *p* = 0.03) and IL-13/IFN-γ ratios (0.04 vs. 0.31; *p* = 0.04).

### 3.4. Biomarker Profiles According to Clinician-Assessed Activity

Total IgG levels were significantly higher in patients with clinically active disease compared to those without (1751.5 mg/dL vs. 1009 mg/dL; *p* = 0.01) (Table 2; Figure 1). No other laboratory or immunological markers reached statistical significance.

### 3.5. Biomarker Profiles According to IgG4-RD Responder Index

Compared with the low IgG4-RD RI group (Table 2; Figure 1), patients with high disease activity showed significantly higher levels of total IgG (1327 mg/dL vs. 975mg/dL; *p* = 0.02), serum IgG4 (117 mg/dL vs. 60.5 mg/dL; *p* = 0.01), and IL-5 (1.63 pg/mL vs. 0.8 pg/mL; *p* = 0.03). There was also a trend toward lower plasmablasts counts (223 vs. 1096 *p* = 0.07), IL-21/IL-5 ratio (0.19 vs. 0.37; *p* = 0.056) and IL-10/IL-5 ratio (2.39 vs. 4.17; *p* = 0.056) in the high IgG4-RD RI group.

### 3.6. Biomarker Correlation with the IgG4-RD Damage Index

The analysis of the same biomarker panel with the IgG4-RD DI, calculated in a subset of 24 patients with at least six months of disease duration, showed no statistically significant associations.

### 3.7. Impact of Immunosuppressive Treatment on Biomarker Levels

All patients received prednisone at disease onset. In the overall cohort (*n* = 35), at the time of blood sampling, 25 patients were receiving prednisone: 21 were on prednisone as the only ongoing treatment, whereas 4 were receiving rituximab concomitantly with prednisone and were excluded from the prednisone analysis to minimize confounding by concomitant therapy. The remaining 10 patients were not receiving glucocorticoids at sampling; of these, 7 were not on any treatment, while 3 were receiving methotrexate and were also excluded from the prednisone analysis to avoid treatment-related bias. After these exclusions, the prednisone comparison included 21 patients with prednisone exclusively and 7 not using any treatment (Table 3).

Compared with non-users, prednisone users had lower IgG4 levels (70 mg/dL vs. 128 mg/dL, *p* = 0.04) and lower C3 levels (mean, 118.19 mg/dL vs. 144.72 mg/dL, *p* = 0.007), with higher CRP levels (8.58 mg/L vs. 1 mg/L, *p* = 0.04) (Table 3). In addition, there was a trend toward lower total IgG concentration among prednisone users (998 mg/dL vs. 1189 mg/dL, *p* = 0.06) (Table 3).

## 4. Discussion

In this study, we demonstrate that a multi-faceted approach is essential for accurately assessing disease activity in IgG4-RD, as different tools reflect unique biological processes. Our data reveal two distinct activity profiles: an IFN-γ-mediated signature corresponding to metabolically active lesions on [18F]-FDG PET/CT and a humoral signature that correlates with the IgG4-RD Responder Index. This distinction underscores the value of using cytokine ratios to understand these complex immune pathways. Importantly, we also show that while these markers do not reflect organ damage, specific biomarkers like plasmablasts can serve as potent pharmacodynamic indicators for targeted therapies such as rituximab and methotrexate, offering a new dimension to treatment monitoring.

We evaluated the clinical utility of novel immunological biomarkers, specifically cytokine expression profiles and plasmablast levels, in a cross-sectional cohort of 35 patients with IgG4-RD. We identified several biomarkers that were significantly associated with disease activity across multiple clinical contexts. Notably, specific cytokine ratios and plasmablast levels demonstrated potential added value beyond conventional markers, offering promising tools for improving disease activity assessment and treatment monitoring in IgG4-RD.

In our cohort, most patients were men in their sixth decade of life, with the retroperitoneal phenotype being the most prevalent subtype. These findings are consistent with previous reports showing a male predominance, middle age at diagnosis, and a higher likelihood of pancreaticobiliary and retroperitoneal involvement in men, whereas head and neck-limited disease is more commonly observed in women [3,4,26].

To date, elevated serum IgG4 has been the most widely used marker of disease activity in IgG4-RD; however, up to 50% of patients with clinically active disease may have normal serum IgG4 levels [27]. When stratifying by serum IgG4 status in the cohort reported by Wallace et al., only 51% of clinically active patients (*n* = 125) exhibited elevated IgG4; nevertheless, those with high IgG4 had a more aggressive phenotype, including older age, higher RI scores, multi-organ involvement, hypocomplementemia, eosinophilia, and raised IgE, compared with active patients whose IgG4 remained normal [22,27]. This finding suggests that, while markedly elevated IgG4 identifies a subgroup with extensive active disease, normal IgG4 does not exclude significant activity. In our cohort, among patients with elevated IgG4, the RI correlated positively with IgG4, total IgG and IL-5. Conversely, in patients with normal IgG4, the RI correlated only with total IgG, highlighting polyclonal hypergammaglobulinemia as a marker of activity when IgG4 is not elevated. In this context, we evaluated novel cytokine ratios alongside established laboratory parameters.

When comparing our cytokine panel with [18F]-FDG PET/CT findings, we observed that peripheral eosinophils were significantly higher in PET-positive patients, consistent with prior reports of mild to moderate peripheral eosinophilia (20–38% of cases) reported in large IgG4-RD cohorts [28]. Among PET-positive patients, only one was receiving glucocorticoid therapy at the time of PET/CT acquisition (prednisone > 10 mg/day). These previous studies have linked eosinophilia with disease duration, greater organ involvement, high RI, elevated serum IgG4, and increased risk of relapse, suggesting it reflects systemic immune activation and ongoing inflammation [29,30,31]. Although not specific to IgG4-RD, elevated eosinophils may support a complementary role in reflecting metabolically active inflammation as detectable by [18F]-FDG PET/CT imaging.

Beyond eosinophil counts, several cytokine ratios were significantly associated with [18F]-FDG PET/CT-defined disease activity, including reduced IL-13/IFN-γ and IL-21/IFN-γ ratios and an elevated IFN-γ/IL-4 ratio. These patterns reflect a shift toward a Th1-predominant immune response and reduced Th2 and Tfh activity in PET-positive patients, which would be in line with recent reviews of IgG4-RD immunopathogenesis describing an imbalance between Th1 cells and Tfh2 response, with local secretion of IL-21 and IFN-γ at lesion sites [10,32]. Th1 cytokines such as IFN-γ promote macrophage activation and suppress fibrotic Th2 responses, whereas Tfh-derived IL-4 and IL-21 are crucial for B cell maturation, antibody production, and IgG4 class-switching [33,34,35]. Moreover, IL-13, produced by CD4+ T cells, mast cells, and ILC2 cells, activates M2 macrophages and contributes to fibrosis by promoting extracellular matrix deposition and tissue fibrosis via the release of profibrotic cytokines including IL-1β and IFN-γ [9,11,13]. In this context, the reduced IL-13/IFN-γ and IL-21/IFN-γ ratios observed in PET-positive subjects suggest a relative predominance of metabolically active, Th1-skewed inflammation over type 2/Tfh-linked signals associated with B cell help and tissue remodeling, consistent with the elevated IFN-γ/IL-4 ratio. Specifically, a lower IL-21/IFN-γ ratio may reflect diminished peripheral Tfh-mediated support for B cell/plasmablast responses (and/or compartmentalization of this axis within affected tissues), whereas a lower IL-13/IFN-γ ratio may be compatible with a less prominent type 2-associated profibrotic signature in this PET-defined active phenotype. Collectively, these findings support the notion that cytokine ratios, rather than absolute levels, better capture the interplay between inflammatory and fibrotic/immunoregulatory processes in IgG4-RD, particularly in metabolically active lesions detected by [18F]-FDG PET/CT [36]. Taken together, these observations align with prior evidence supporting a pathogenic Tfh–B cell axis in IgG4-RD, in which IL-21-related Tfh activity promotes plasmablast expansion and humoral activation, providing a plausible biological framework for interpreting the reduced IL-21/IFN-γ (and IL-13/IFN-γ) ratios in PET-positive disease [36].

In our cohort, the clinician’s judgment of disease activity proved highly reliable, although among all laboratory tests only total IgG levels differed significantly between active and inactive patients, reflecting its value as a surrogate for polyclonal hypergammaglobulinemia driven in part by the IgG4 subclass [37]. By contrast, standard interleukin measurements and their ratios failed to distinguish activity states, mirroring systematic-review findings that cytokine profiles correlate poorly with clinical flares in IgG4-RD [12]. The exclusive significance of total IgG likely stems from its incorporation of IgG4 elevations within the broader immunoglobulin pool, as previously described in IgG4-RD cohorts [38]. This suggests that clinicians may intuitively rely on indicators of global humoral activation when evaluating disease activity in IgG4-RD. This finding underscores a potential disconnect between the immunological complexity of IgG4-RD and the practical tools currently guiding clinical decision-making. It also reinforces the need for accessible, reliable biomarkers that align more closely with real-world clinical assessments to improve disease monitoring.

Moreover, higher IgG4-RD RI was accompanied by increased humoral markers, including total IgG and IgG4. In keeping with current immunopathogenic concepts, this pattern should not be interpreted as evidence of a purely Th2-driven disorder; instead, active IgG4-RD appears to display a dominant Th1-oriented inflammatory milieu with an additional Th2/regulatory component that may amplify humoral activation during flares. These findings are consistent with Wallace et al., where increased concentrations of these markers have been shown to have strong correlations with RI scores and clinical disease activity [13]. However, although the IgG4-RD RI remains the most widely used tool to quantify disease activity, it has important limitations. Firstly, the inclusion of serum IgG4 in the score introduces circularity, as any correlation between RI and IgG4 is partly tautological. Secondly, it can lead to underestimation in patients with active disease but normal IgG4 levels, and overestimation among those with minimal symptoms but elevated IgG4. Finally, the RI does not always distinguish active inflammation from irreversible fibrotic damage: although validation studies recommended separate “activity” and “damage” scores per organ, this distinction is often subtle in clinical practice [21]. Consequently, RI interpretation must always be integrated with comprehensive clinical assessment, imaging, and histology.

In IgG4-RD active disease as assessed with the RI score, we also observed a significant elevation of IL-5—an essential Th2 secreted cytokine for eosinophil differentiation and survival and accompanying trends toward reduced IL-10/IL-5 and IL-21/IL-5 ratios, indicating a predominance of Th2 over regulatory or Tfh and an immune environment favoring eosinophil-driven inflammation and B cell class-switching [28]. IL-5 has previously been reported to be elevated in patients with IgG4-RD [38,39,40], with levels increasing in parallel with advancing clinical stage, and significantly higher in patients with a poor prognosis [41,42]. Finally, we observed a trend toward lower circulating plasmablast levels in patients with high RI scores. Although this appears counterintuitive given the established role of plasmablasts as disease activity markers independent of IgG4 serum levels, it may reflect prior or concomitant immunosuppressive therapy or interindividual variability in B cell activation dynamics [13,43].

Among conventional markers, both total IgG and IgG4 levels were significantly elevated in active patients (*p* = 0.022 and *p* = 0.045, respectively), and IgG4 also correlated with physician-assessed activity (*p* = 0.02). However, neither CRP nor ESR showed significant associations with activity, reinforcing their limited sensitivity in this setting.

Notably, none of the immunological biomarkers, including cytokine ratios and plasmablast levels, correlated with the IgG4-RD Damage Index (DI). This likely reflects the distinct nature of DI as a cumulative, largely irreversible measure of organ damage, in contrast to the dynamic immune activity captured by circulating biomarkers. These findings reinforce the concept that active inflammation and structural damage represent biologically and clinically separate domains in IgG4-RD, each requiring tailored assessment tools [22].

Regarding pharmacological treatment, prednisone use in our cohort was associated with statistically significant reductions in IgG4 and C3 levels. The decline in serum IgG4 is consistent with previous studies showing that corticosteroid therapy reliably suppresses IgG4 production in most patients with IgG4-RD [32,44]. In contrast, the significant reduction in C3 may seem counterintuitive, as low complement levels are negatively correlated with indicators of disease activity and low levels normalize quickly after immunosuppressive therapy [45]. This apparent discrepancy may reflect persistently high pre-treatment disease activity leading to ongoing complement consumption, or residual immune activation despite corticosteroid therapy.

A key strength of this study is its comprehensive and clinically relevant design. Our multi-modal assessment, integrating real-world clinical judgment, the IgG4-RD Responder Index, [18F]-FDG PET/CT imaging, and response to therapy, allowed for a granular stratification of biomarker performance across different clinical scenarios. This was complemented by an extensive analysis of a broad panel of biomarkers. Notably, our focus on cytokine ratios, rather than absolute levels, offered deeper insight into the underlying immune polarization and helped identify distinct functional profiles, such as inflammatory versus fibrotic states, which are not captured by conventional markers. Collectively, we believe this approach advances our understanding of the disease’s complex pathophysiology and lays the groundwork for developing more targeted biomarkers in the future. A further strength is the prospective nature of our data collection for this cross-sectional analysis. Clinical assessments and biological samples were obtained contemporaneously during the same patient visit. This methodology minimizes the latency between clinical evaluation and laboratory measurement, thereby strengthening the validity of the observed associations between biomarker profiles and disease states.

We acknowledge several limitations in this study. First, the modest sample size and single-center design may limit the generalizability of our findings. However, this is partially mitigated by our status as a national referral center with extensive multidisciplinary collaboration, which ensures a high quality of data and diagnostic certainty. Second, the clinical heterogeneity of our patient cohort, which included varied phenotypes and treatment histories, could be viewed as a limitation. Conversely, we consider this a strength, as it reflects the complex nature of IgG4-RD in a real-world setting. Evaluating biomarkers in such a diverse group is crucial for assessing their utility in the target population where they are ultimately intended for use. Finally, while our sample size may lack the statistical power to detect more subtle associations, the fact that our key findings reached statistical significance suggests they are robust, although they undoubtedly warrant validation in larger, multi-center cohorts.

## 5. Conclusions

This study identified total IgG, serum IgG4, IL-5 levels, and specific cytokine ratios as potential indicators of disease activity in IgG4-RD, including [18F]-FDG PET/CT findings and the IgG4-RD RI. Moreover, elevated eosinophil counts and a Th1-skewed cytokine profile (e.g., increased IFN-γ/IL-4 ratio) were associated with active metabolic lesions on [18F]-FDG-PET/CT, supporting their value in detecting clinically relevant inflammation. Although plasmablast levels were previously proposed as sensitive markers of disease activity, they were slightly lower in patients with high RI scores and in those receiving immunosuppressive treatment with methotrexate and rituximab, suggesting that their interpretation must consider treatment status and immune modulation. Overall, these findings suggest that cytokine-based immunophenotyping, in combination with conventional markers and imaging, can enhance assessment of disease activity and guide personalized therapeutic strategies in IgG4-RD. Future longitudinal studies in larger cohorts are warranted to validate their prognostic value and define their role in clinical decision-making.

## Figures and Tables

**Table 1:** Demographic, clinical, radiologic, and histologic characteristics of the study cohort.

Category	Variable	IgG4-RD Patients *n* = 35
**Demographics, *n* (%)**	Age, median (P25–P75)	63.0 (52–72)
	Sex (men)	27 (77.1)
**Diagnostic Criteria, *n* (%)**	Okazaki	23 (65.7)
	Umehara	
	*Possible*	20 (57.1)
	*Probable*	2 (5.7)
	*Definite*	10 (28.6)
	ACR/EULAR	23 (65.7)
**Clinical Scores and Indices, *n* (%)**	Responder Index, mean (SD)	4.83 (4.0)
	>4	19 (54.3)
	Clinically assessed active disease	12 (34.3)
	Damage Control Index, mean (SD)	3.09 (1.5)
**Organ Involvement, *n* (%)**	Increased organ size	33 (94.3)
	Glandular	
	*>1*	3 (8.8)
	*>2*	3 (8.8)
	Pancreas	
	*Pancreas and biliary tract*	7 (20.0)
	*Diffuse pancreatic enlargement with* *capsule-like rim of low density*	2 (5.7)
	*Diffuse pancreatic enlargement* *(loss of lobulation)*	1 (2.9)
	Renal	
	*Bilateral low-density areas in the renal cortex*	10 (28.6)
	*Hydronephrosis*	5 (14.3)
	*Hypocomplementemia*	4 (11.4)
	*Thickening of the renal pelvis/soft tissue*	3 (8.6)
	Retroperitoneal	
	*Peripheral or anterolateral soft tissue around the infrarenal aorta or iliac arteries*	12 (34.3)
	*Diffuse thickening of the abdominal aortic wall*	8 (22.9)
	Thorax	
	*Peribronchovascular and septal thickening*	5 (14.3)
	*Soft tissue resembling a paravertebral band*	2 (5.7)
	Aneurism	13 (37.1)
**Wallace Subtype Classification, *n* (%)**	Group 1. Pancreato-biliary	8 (22.9)
	Group 2. Retroperitoneum and aorta	21 (60.0)
	Group 3. Systemic Mikulicz	5 (14.3)
	Group 4. Head and neck	1 (2.9)
**Systemic Symptoms, *n* (%)**	Low back pain	8 (22.9)
	Toxic syndrome	7 (20.0)
	Fever	5 (14.3)
**Neurological and Head Involvement, *n* (%)**	Hypophysitis	1 (2.9)
	Pachymeningitis	1 (2.9)
	Orbital pseudotumor	0 (0.0)
	Uveal involvement	0 (0.0)
**Imaging Findings, *n* (%)**	Activity based on positive PET	8 (28.6)
	CT	14 (40.0)
	*Aorta involvement*	3 (21.4)
	*Retroperitoneum involvement*	3 (21.4)
**Histological Findings, *n* (%)**		11 (31.4)
	Plasmatic cells	10 (90.9)
	Lymphocytic infiltration	10 (90.9)
	Fibrosis	4 (36.4)
	Phlebitis	3 (27.3)
**Serologic biomarkers**	IgG (P25–P75)	1168 (954–1808.5) (mg/dL)
	IgG4 (P25–P75)	91 (33–138) (mg/dL)
	Eosinophil count (P25–P75)	0.16 (0.08–0.26) × 10^3^ cells/μL)
	CRP (P25–P75)	2.5 (1–5.05) (mg/L)
	ERS (P25–P75)	9 (0–24) (mm/h)

ACR/EULAR, American College of Rheumatology/European League Against Rheumatism; CT, computed tomography; P25–P75, 25th to 75th percentile (interquartile range); PET, 18F-fluorodeoxyglucose positron emission tomography; SD, standard deviation.

**Table 2:** Levels of analytical and serological biomarkers with disease activity, measured as PET positivity, physician judgment, and Responder Index (RI).

Category	Variable	PET + (*n* = 8)	PET − (*n* = 20)	*p*-Value	Clinician Assessment: Active (*n* = 12)	Clinician Assessment: Not Active (*n* = 23)	*p*-Value	RI ≥ 4 (*n* = 19)	RI < 4 (*n* = 16)	*p*-Value
**Cellular** **markers, median (IQR)**	Plasmablasts, cells/μL	1226 (141.75–2024.75)	202.50 (0–1121)	0.22	401 (0–1219)	722 (0–1606)	0.71	223 (0–1054)	1096 (0–2955)	0.07
	Plasmablasts/IgG4	0.77 (0–10.06)	8.04 (3.48–16.85)	0.31	4.4 (0–32.52)	2.61 (0–10.5)	0.63	0.97 (0–7.26)	19.30 (0–43.09)	0.08
**Hematologic markers, median (IQR)**	Eosinophils. ×10^3^ cells/μL	0.24 (0.17–0.39)	0.15 (0.08–0.21)	**0.03**	0.15 (0.09–0.23)	0.16 (0.08–0.34)	0.4	0.15 (0.08–0.23)	0.19 (0.10–0.37)	0.18
	Hemoglobin, g/dL	13 (11.88–14.55)	14.4 (13.55–15)	0.175	14.1 (12.65–15.45)	14.3 (13.45–14.8)	0.7	14.2 (13.20–14.95)	14.35 (13.30–14.95)	0.65
**Acute-phase markers, median (IQR)**	CRP, mg/L	2.55 (1.62–5.22)	3.15 (1–6.35)	0.78	1.75 (1–5.15)	2.5 (1–5)	0.96	2.50 (1–5.3)	2.40 (1–4.28)	0.80
	ESR, mm/h	9 (0–22)	11.5 (4–24.75)	0.6	11 (4–13)	6.5 (0–37)	0.64	10 (0–18.50)	6 (1–31.00)	0.84
**Complement system, mean (SD)**	C3, mg/dL	130.25 (25.8)	125.27 (32.2)	0.71	135.17 (26.6)	118.07 (28.3)	0.12	129.93 (27.3)	120.55 (30.1)	0.41
	C4, mg/dL	26.10 (11.3)	26.61 (6.3)	0.88	28.12 (8.7)	25.36 (6.9)	0.37	27.36 (8.2)	25.65 (7.4)	0.58
**Humoral markers, median (IQR)**	IgG, mg/dL	1759.5 (920.5–2046.5)	1003.5 (931–1348.25)	0.33	1751.5 (1131.25–2046.5)	1009 (933–1313)	**0.01**	1327 (1051–1919.5)	975 (878–1366.25)	**0.02**
	IgG4, mg/dL	103.5 (47.25–132.50)	82 (37.25–118.75)	0.74	114 (81.5–176.25)	75 (37–114)	0.2	117 (72.5–226)	60.5 (32–97)	**0.01**
	IgG4/IgG	0.06 (0.05–0.07)	0.07 (0.03–0.10)	0.57	0.06 (0.04–0.1)	0.07 (0.04–0.1)	0.74	0.10 (0.08)	0.06 (0.03)	0.21
**Cytokines, median (IQR)**	INF-γ, pg/mL	5.95 (3.91–9.71)	4.29 (2.17–7.38)	0.19	4.32 (2.79–8.13)	5.21 (2.25–7.72)	0.64	4.68 (2.75–8.64)	4.58 (2.14–7.47)	0.46
	IL-10, pg/mL	1.88 (0.56–12.64)	5.64 (1.65–9.66)	0.27	1.28 (0.56–8.15)	3.99 (1.58–6.04)	0.43	2.32 (0.67–7.51)	3.59 (1.37–5.79)	0.99
	IL-13, pg/mL	0.23 (0.17–1.29)	1.94 (0.23–9.34)	0.16	0.23 (0.23–9.34)	1.75 (0.23–2.94)	0.87	1.85 (0.23–4.96)	0.23 (0.23–2.32)	0.21
	IL-1β, pg/mL	0.57 (0.34–1.09)	0.42 (0.14–0.83)	0.52	0.49 (0.33–1.08)	0.49 (0.14–0.84)	0.52	0.55 (0.32–1.21)	0.44 (0.14–0.67)	0.33
	IL-21, pg/mL	0.21 (0.15–0.44)	0.26 (0.14–0.44)	0.98	0.21 (0.14–0.46)	0.25 (0.14–0.4)	1	0.3 (0.14–0.47)	0.21 (0.14–0.39)	0.58
	IL-4, pg/mL	2.67 (1.12–5.41)	6.83 (2.62–25.91)	0.17	3.69 (1.12–18.40)	4.59 (1.25–9.42)	0.89	4.24 (1.79–20.2)	4.31 (1.12–9.02)	0.55
	IL-5, pg/mL	1.35 (0.69–2.21)	1.58 (0.60–2.63)	1	1.85 (0.7–2.85)	1.01 (0.41–2.00)	0.15	1.63 (0.97–2.66)	0.8 (0.27–2)	**0.03**
**Cytokine** **ratios, median (IQR)**	INF-γ/IL-10	3.76 (0.99–7.39)	1.3 (0.36–2.14)	0.14	2.93 (1.22–6.29)	1.5 (0.51–2.84)	0.26	1.81 (0.99–4.8)	1.48 (0.43–2.67)	0.46
	IL-13/INF-γ	0.04 (0.02; 0.34)	0.31 (0.11–1.01)	**0.04**	0.1 (0.06–1.01)	0.2 (0.08–0.77)	0.82	0.29 (0.07–0.91)	0.15 (0.04–0.53)	0.45
	INF-γ/IL-1β	9.14 (6.28–20.78)	8.84 (6.88–14.34)	0.82	7.34 (6.44–11.57)	9.24 (6.2–14.82)	0.52	8.37 (6.33–15.21)	9.05 (6.28–14.34)	0.72
	IL-21/INF-γ	0.03 (0.02–0.06)	0.07 (0.04–0.10)	**0.03**	0.06 (0.03–0.07)	0.07 (0.03–0.09)	0.48	0.06 (0.03–0.07)	0.07 (0.03–0.09)	0.46
	INF-γ/IL-4	3.46 (1.06–4.57)	0.98 (0.37–1.32)	**<0.001**	1.22 (0.5–2.79)	1.11 (0.48–1.63)	0.67	1.11 (0.51–1.69)	1.18 (0.44–2.22)	0.78
	INF-γ/IL-5	3.44 (2.69–8.45)	2.98 (1.94–5.48)	0.38	3.44 (2.75–4.45)	3.45 (2.38–12.2)	0.67	3.03 (2.65–3.83)	5.76 (2.35–14.35)	0.16
	IL-13/IL-10	0.41 (0.04–0.46)	0.41 (0.14–1.15)	0.4	0.41 (0.37–0.81)	0.41 (0.12–1.04)	0.79	0.49 (0.33–1.19)	0.41 (0.09–0.81)	0.22
	IL-10/IL-1β	4.7 (1.23–18.87)	7.44 (4.09–38.25)	0.24	4.06 (1.37–6.46)	6.6 (3.84–25.5)	0.22	4.69 (2.47–11.59)	6.84 (3.74–18.87)	0.45
	IL-21/IL-10	0.1 (0.01–0.32)	0.07 (0.02–0.16)	0.94	0.16 (0.05–0.27)	0.09 (0.03–0.18)	0.48	0.08 (0.04–0.24)	0.1 (0.03–0.2)	0.93
	IL-10/IL-4	0.68 (0.37–1.79)	0.48 (0.22–1.25)	0.51	0.48 (0.24–0.81)	0.5 (0.25–1.32)	0.59	0.47 (0.23–0.82)	0.5 (0.26–1.25)	0.45
	IL-10/IL-5	1.1 (0.50–12.00)	3.91 (2.04–9.94)	0.38	1.6 (0.7–4.79)	4.1 (1.19–10.06)	0.9	2.39 (0.67–6.69)	4.17 (1.21–14.27)	0.056
	IL-13/IL-1β	1.11 (0.19–3.13)	2.29 (1.57–10.77)	0.14	1.64 (0.56–6.36)	1.64 (1.50–5.82)	0.65	2.50 (1.07–7.32)	1.64 (0.97–3.81)	0.55
	IL-13/IL-21	1.53 (0.72–4.89)	3.40 (1.64–18.47)	0.17	1.64 (1.49–13.64)	3.3 (1.64–9.54)	1	4.32 (1.64–16.6)	1.64 (1.18–5.47)	0.18
	IL-13/IL-4	0.15 (0.05–0.29)	0.21 (0.14–0.29)	0.43	0.21 (0.1–0.36)	0.21 (0.15–0.29)	0.79	0.21 (0.14–0.43)	0.21 (0.12–0.24)	0.32
	IL-13/IL-5	0.24 (0.08–1.09)	1.6 (0.32–5.29)	0.07	0.89 (0.18–3.45)	1.5 (0.46–2.18)	0.63	1.50 (0.26–2.94)	1.04 (0.32–2.05)	0.79
	IL-21/IL-1β	0.55 (0.3–0.95)	0.73 (0.48–1)	0.26	0.47 (0.4–0.70)	0.73 (0.48–1)	0.21	0.49 (0.41–0.78)	0.83 (0.49–1)	0.14
	IL-1β/IL-4	0.13 (0.11–0.45)	0.07 (0.04–0.13)	0.06	0.13 (0.06–0.29)	0.12 (0.05–0.14)	0.38	0.1 (0.04–0.23)	0.12 (0.07–0.13)	1
	IL-1β/IL-5	0.46 (0.22–0.65)	0.29 (0.15–1.02)	0.74	0.5 (0.14–0.67)	0.35 (0.23–1.13)	0.66	0.36 (0.15–0.72)	0.53 (0.28–1.17)	0.3
	IL-21/IL-4	0.08 (0.03–0.13)	0.05 (0.02–0.11)	0.56	0.07 (0.02–0.13)	0.07 (0.03–0.12)	0.78	0.04 (0.02–0.13)	0.08 (0.03–0.12)	0.67
	IL-21/IL-5	0.17 (0.08–0.27)	0.21 (0.11–0.52)	0.44	0.17 (0.08–0.42)	0.23 (0.15–0.52)	0.25	0.19 (0.09–0.36)	0.37 (0.17–0.67)	0.056
	IL-4/IL-5	3.67 (0.93–4.60)	6 (2.62–28.48)	0.07	4.68 (1.46–7.44)	4.54 (2.74–19.49)	0.38	3.50 (1.95–9.18)	4.71 (3.83–18.75)	0.24

C3, complement component 3; C4, complement component 4; CRP, C-reactive protein; ESR, erythrocyte sedimentation rate; IFN-γ, interferon gamma; IgG, immunoglobulin G; IgG4, immunoglobulin G4; IL, interleukin; PET, 18F-fluorodeoxyglucose positron emission tomography/CT scan; RI, Responder Index; IQR, interquartile ranges. Mann–Whitney U test used for all variables except for complement system in which we used *t*-test.

**Table 3:** Levels of analytical and serological biomarkers by treatment received.

Category	Variable	PREDNISONE		
		Yes (*n* = 21)	No (*n* = 7)	*p*-Value
**Cellular markers, median (IQR)**	Plasmablasts, cells/μL	416 (0–1139)	579 (571–1638)	0.12
	Plasmablasts/IgG4	4.4 (0–10.95)	2.67 (0.12–35.2)	0.87
**Hematologic markers, median (IQR)**	Eosinophils, ×10^3^ cells/μL	0.15 (0.08–0.2)	0.08 (0.08–0.16)	0.166
	Hemoglobin. g/dL	14.3 (13.6–14.9)	14.4 (14.20–14.6)	0.72
**Acute-phase markers, median (IQR)**	CRP, mg/L	8.58 (16.2)	1 (1–1.4)	**0.04**
	ESR, mm/h	20.80 (22.5)	11 (7.50–13.00)	0.80
**Complement system, mean (SD)**	C3, mg/dL	118.19 (26.2)	144.72 (17.1)	**0.007**
	C4, mg/dL	27.02 (6.4)	31.1 (4.5)	0.106
**Humoral markers, median (IQR)**	IgG, mg/dL	998 (887–1275)	1189 (998.5–1831)	0.06
	IgG4, mg/dL	70 (26–104)	128 (32–186)	**0.04**
	IgG4/IgG	0.06 (0.02–0.09)	0.03 (0.02–0.182)	0.69
**Cytokines, median (IQR)**	INF-γ, pg/mL	5.21 (2.28–8.22)	5.4 (3.24–8.88)	0.63
	IL-10, pg/mL	4.82 (1.64–6.51)	2.07 (0.73–6.18)	0.8
	IL-13, pg/mL	1.75 (0.23–4.63)	3.13 (1.32–3.40)	0.12
	IL-1β, pg/mL	0.44 (0.14–0.72)	0.66 (0.38–0.99)	0.31
	IL-21, pg/mL	0.3 (0.14–0.48)	0.26 (0.20–0.32)	0.91
	IL-4, pg/mL	4.48 (1.37–16.58)	9.65 (6.17–13.34)	0.22
	IL-5, pg/mL	1.54 (0.42–2.36)	2.02 (0.81–2.65)	0.59
**Cytokine ratios, median (IQR)**	INF-γ/IL-10	1.49 (0.58–2.30)	1.81 (1.32–3.31)	0.88
	IL-13/INF-γ	0.20 (0.06–0.98)	0.34 (0.21–1.03)	0.17
	INF-γ/IL-1β	9.90 (6.36–15.79)	8.5 (7.34–9.57)	0.6
	IL-21/INF-γ	0.07 (0.04–0.11)	0.05 (0.045–0.069)	0.9
	INF-γ/IL-4	1.11 (0.48–1.42)	1.16 (0.64–1.22)	0.5
	INF-γ/IL-5	0.03 (2.15–6.00)	3.29 (2.79–9.9)	0.46
	IL-13/IL-10	0.41 (0.14–1.12)	1.06 (0.47–1.26)	0.06
	IL-10/IL-1β	0.6 (3.85–34.43)	3.67 (3.05–5.63)	0.39
	IL-21/IL-10	0.09 (0.03–0.19)	0.2 (0.12–0.31)	0.41
	IL-10/IL-4	0.47 (0.27–1.18)	0.47(0.23–0.57)	0.34
	IL-10/IL-5	3.62 (1.24–9.82)	3.62 (1.72–5.05)	0.88
	IL-13/IL-1β	1.64 (1.64–7.33)	3.64 (1.80–6.69)	0.3
	IL-13/IL-21	3.3 (1.64–13.90)	9.60 (5.56–13.64)	0.91
	IL-13/IL-4	0.21 (0.17–0.29)	0.3 (0.23–0.2)	0.9
	IL-13/IL-5	1.5 (0.35–2.61)	2.01 (0.43–2.30)	0.09
	IL-21/IL-1β	0.84 (0.52–1)	0.67 (1.01–4.8)	0.08
	IL-1β/IL-4	0.1 (0.04–0.12)	0.12 (0.07–0.16)	0.7
	IL-1β/IL-5	0.35 (0.21–0.61)	0.6 (0.33–1.14)	0.39
	IL-21/IL-4	0.07 (0.03–0.12)	0.03 (0.02–0.10)	0.59
	IL-21/IL-5	0.23 (0.15–0.50)	0.34 (0.13–0.4)	0.81
	IL-4/IL-5	4.59 (2.80–20.98)	5.87 (2.91–9.3)	0.78

C3, complement component 3; C4, complement component 4; CRP, C-reactive protein; ESR, erythrocyte sedimentation rate; IFN-γ, interferon gamma; IgG, immunoglobulin G; IgG4, immunoglobulin G4; IL, interleukin; IQR, interquartile ranges. Mann–Whitney U test used for all variables except for complement system in which we used *t*-test.

## Data Availability

The data presented in this study are available from the corresponding author upon reasonable request. Due to the retrospective clinical nature of the study and to protect patient privacy, the data are not publicly available.

## Abbreviations

The following abbreviations are used in this manuscript:

ACR/EULAR	American College of Rheumatology/European League Against Rheumatism
C3	Complement component 3
C4	Complement component 4
CRP	C-reactive protein
CT	Computed tomography
ESR	Erythrocyte sedimentation rate
IFN-γ	Interferon gamma
IgG	Immunoglobulin G
IgG4	Immunoglobulin G4
IL	Interleukin
P25–P75	25th to 75th percentile (interquartile range)
PET	18F-fluorodeoxyglucose positron emission tomography
PET/CT	18F-fluorodeoxyglucose positron emission tomography/computed tomography
RI	Responder Index
SD	Standard deviation
